# In situ gastroepiploic artery versus I-composite right internal thoracic artery radial artery for severe right coronary artery stenosis in off-pump coronary artery bypass grafting

**DOI:** 10.1016/j.xjon.2025.04.009

**Published:** 2025-05-02

**Authors:** Hideki Isa, Ryohei Ushioda, Baku Takahashi, Dit Yoongtong, Boonsap Sakboon, Jaroen Cheewinmethasiri, Hiroyuki Kamiya, Nuttapon Arayawudhikul

**Affiliations:** a Cardiovascular and Thoracic Surgery Unit, Department of Surgery, Lampang Hospital, Lampang, Thailand; b Department of Cardiac Surgery, Asahikawa Medical University, Asahikawa, Japan

**Keywords:** off-pump coronary artery bypass grafting, gastroepiploic artery, I-composite graft, right internal thoracic artery, radial artery, right coronary artery

## Abstract

**Objective:**

This study compared the I-composite graft comprising the right internal thoracic artery and radial artery with the gastroepiploic artery in off-pump coronary artery bypass grafting for severe right coronary artery stenosis.

**Methods:**

This study included 78 and 141 patients who underwent right internal thoracic artery-radial artery and gastroepiploic artery grafting, respectively, for off-pump coronary artery bypass grafting between April 2011 and June 2024. Propensity score matching was conducted, and postoperative outpatient follow-up was performed.

**Results:**

Propensity score matching resulted in 65 patients in each group. Preoperative characteristics, operative time, and the number of arterial revascularizations and distal anastomoses did not differ significantly between the groups. However, more graft conduits were used in the right internal thoracic artery-radial artery group. Short-term postoperative outcomes were similar, except for a significantly higher early extubation rate in the right internal thoracic artery-radial artery group. During a median follow-up of 5.0 years, overall survival did not differ significantly between the groups. However, the gastroepiploic artery group had a significantly lower long-term freedom from major adverse cardiac and cerebrovascular events, particularly heart failure requiring hospitalization. Multivariate analysis identified a history of peripheral arterial disease as a significant risk factor for overall mortality, whereas the type of right coronary artery graft was not.

**Conclusions:**

Using the gastroepiploic artery as a direct conduit for severe right coronary artery stenosis appears to be a promising option when selecting an arterial graft, particularly in improving major adverse cardiac or cerebrovascular events-free survival.

## Graphical Abstract

**Figure:**
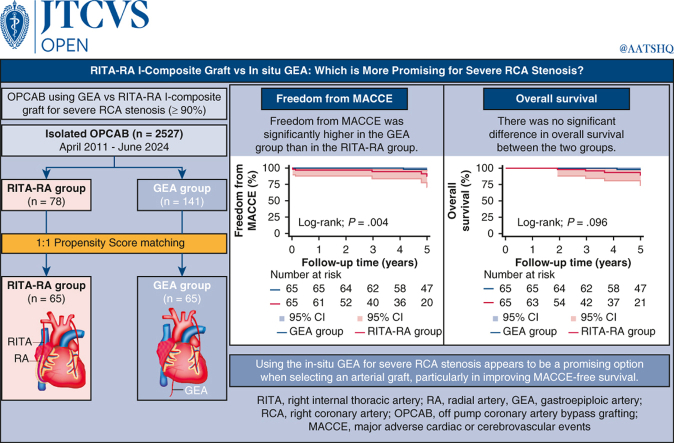

**Figure:**
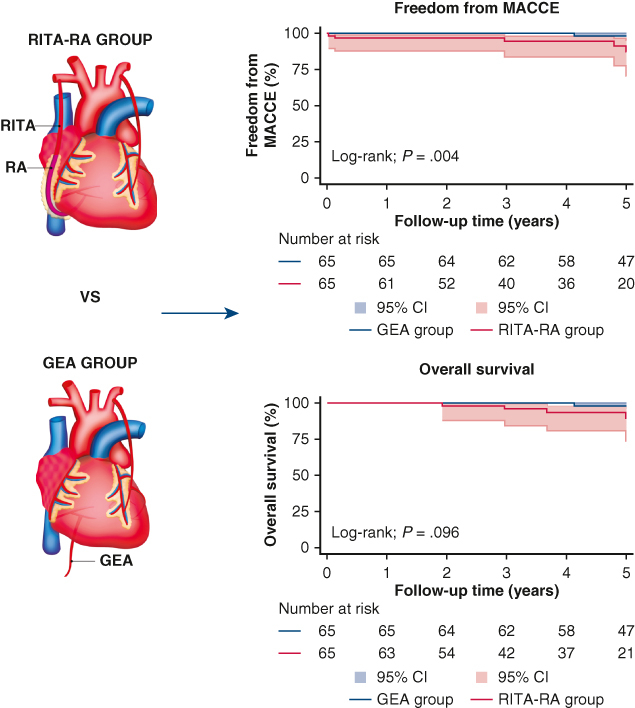
In situ GEA graft over RITA-RA I-composite graft for severe RCA stenosis in OPCAB.

**Central Message**

The in situ GEA may be a promising option for arterial grafting to severe RCA stenosis in OPCAB, particularly in improving MACCE-free survival.

**Perspective**

The optimal arterial graft for severe RCA stenosis remains controversial. This study compared the RITA-RA I-composite graft and the GEA in patients undergoing OPCAB. Findings suggest that the in situ GEA may be a more appropriate graft than the RITA-RA I-composite graft, especially for MACCE-free survival.

Multiarterial coronary artery bypass grafting (CABG) improves long-term survival.^1^, ^2^, ^3^ Although the internal thoracic artery (ITA) is universally regarded as the optimal graft for the left anterior descending artery, there is no consensus on the optimal arterial graft for the right coronary artery (RCA). The right ITA (RITA) graft to the RCA has a 10-year patency of approximately 84%, which is lower than that of other coronary territories.^4^ Some reports recommend using the radial artery (RA) due to risks associated with the RITA such as deep sternal wound infections.^5^

The no-touch aorta technique (NAT) significantly reduces the incidence of stroke and other complications,^6^, ^7^, ^8^ but requires a new inflow source for the RA. To address this, our department uses an I-composite graft combining the RITA and RA for severe RCA stenosis in off-pump coronary artery bypass grafting (OPCAB). This study evaluated the effectiveness of this graft compared with the right gastroepiploic artery (GEA), another potential option for NAT.

## Materials and Methods

### Patients

This study was approved by the institutional review board of Lampang Hospital (No. 028/68; February 7, 2025), which waived the requirement for oral and written informed consent due to its retrospective nature. Data from patients who underwent isolated OPCAB between April 2011 and June 2024 were analyzed. Patients who underwent either RITA-RA or GEA grafting for ≥90% stenosis of the RCA were included, excluding those with poor preoperative conditions (eg, emergency or salvage cases, Society of Thoracic Surgeons risk score >20, or European System for Cardiac Operative Risk Evaluation II score >20). Graft selection was based on patient age, comorbidities, and the surgeon's discretion.

**Figure 1:**
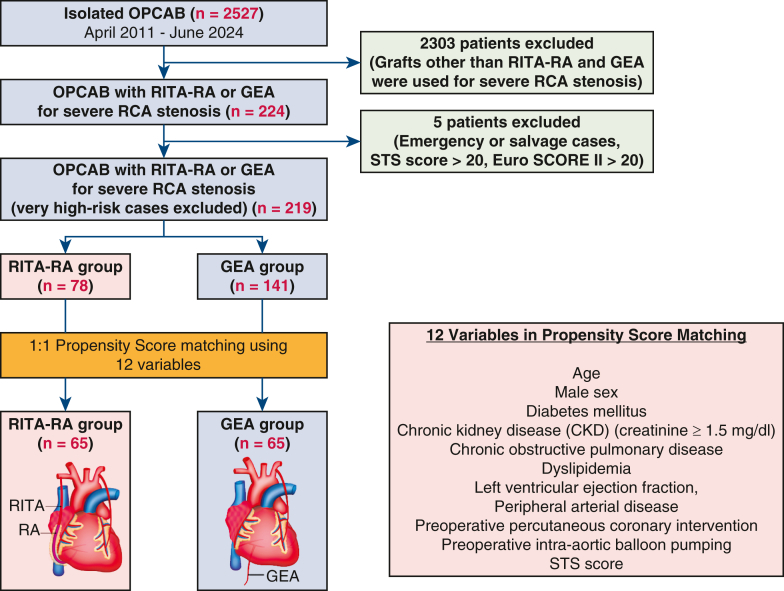
Consolidated Standards for Reporting Trials-style diagram of showing inclusion/exclusion of the patient groups. *OPCAB*, Off-pump coronary artery bypass grafting; *RITA-RA*, right internal thoracic artery-radial artery; *GEA*, gastroepiploic artery; *STS*, Society of Thoracic Surgeons; *EuroSCORE*, European System for Cardiac Operative Risk Evaluation.

Propensity score matching (PSM) (1:1) was then performed based on 12 covariates of preoperative clinical characteristics (Figure 1). Preoperative characteristics, perioperative details, and postoperative outcomes were compared between groups. Postoperative follow-up was conducted every 6 months, with follow-ups via telephone calls for patients who missed appointments. Dual antiplatelet therapy was prescribed at discharge.

### Surgical Techniques

All the patients underwent OPCAB following previously established techniques.^9^ The RA was harvested by combining NAT and low-energy cauterization. The I-composite graft was created via end-to-end anastomosis of the RA to the RITA using 8-0 polypropylene. After anastomosis, the RA component of the I-composite graft was dilated gradually by blood pressure. To harvest the GEA, a median incision approximately 5 cm larger than standard was made. After opening the peritoneal cavity, the GEA was exposed and assessed for size and calcification. Harvesting was discontinued if the GEA was too small or if severe and widespread atherosclerotic lesions were detected. A skeletonized GEA with favorable properties was harvested using a Harmonic FOCUS device (Ethicon Endo-Surgery Inc). After systemic heparinization, the GEA was clipped, cut distally, and treated with phosphodiesterase III inhibitor before being clipped again at the cut edge.

### Data Collection and Statistical Analysis

Data were collected from electronic medical records and analyzed using EZR (Saitama Medical Center, Jichi Medical University),^10^ a graphical user interface for R (R Foundation for Statistical Computing). Values are expressed as mean ± SD for continuous variables and n (%) for categorical variables. The Kolmogorov-Smirnov test assessed normality. The *t* test was used for normally distributed continuous variables, whereas the Mann-Whitney *U* test and Wilcoxon signed-rank test were used for nonnormal data in the entire and matched cohorts, respectively. Categorical variables were analyzed using the χ^2^ or Fisher exact test in the entire cohort and McNemar test in the matched cohort unless highly imbalanced with few discordant pairs.

PSM was adjusted for prespecified clinically relevant baseline characteristics that could be confounding variables. The PS was calculated using logistic regression models, including 12 covariables (age, male sex, diabetes mellitus, chronic kidney disease [CKD] [creatinine ≥1.5 mg/dL)], chronic obstructive pulmonary disease, dyslipidemia, left ventricular ejection fraction, peripheral arterial disease, preoperative percutaneous coronary intervention, preoperative intra-aortic balloon pumping [IABP], and Society of Thoracic Surgeons risk score). Multicollinearity was assessed using the variance inflation factor. All variance inflation factor values were <5, indicating no multicollinearity concerns. Patients were matched 1:1 using the nearest-neighbor matching method with a caliper width of 0.15 of the SD of the logit of the estimated PS. The Kaplan-Meier method was used to determine the overall survival and freedom from major adverse cardiac or cerebrovascular events (MACCE), which was defined as heart failure requiring hospitalization, graft failure requiring reintervention, cardiac death, and cerebral stroke and bleeding. Univariable or multivariable Cox proportional hazards regression analyses were used to identify independent prognostic factors of long-term MACCE and overall death. Our department includes 4 surgeons, 1 of whom (author N.A.) performed approximately 70% of the cases in this cohort. To evaluate the potential influence of surgeon-related bias, long-term MACCE and overall death were compared between Dr Arayawudhikul and the other surgeons in both univariable and multivariable analyses. To further assess potential time-related bias in graft selection, the study period was divided into 2 operative periods (2011-2017 and 2018-2024), and graft usage between groups was compared.

### End Points

The primary end point was the occurrence of a composite of death and MACCE. The secondary end points included the individual events of the primary outcome.

## Results

**Figure 2:**
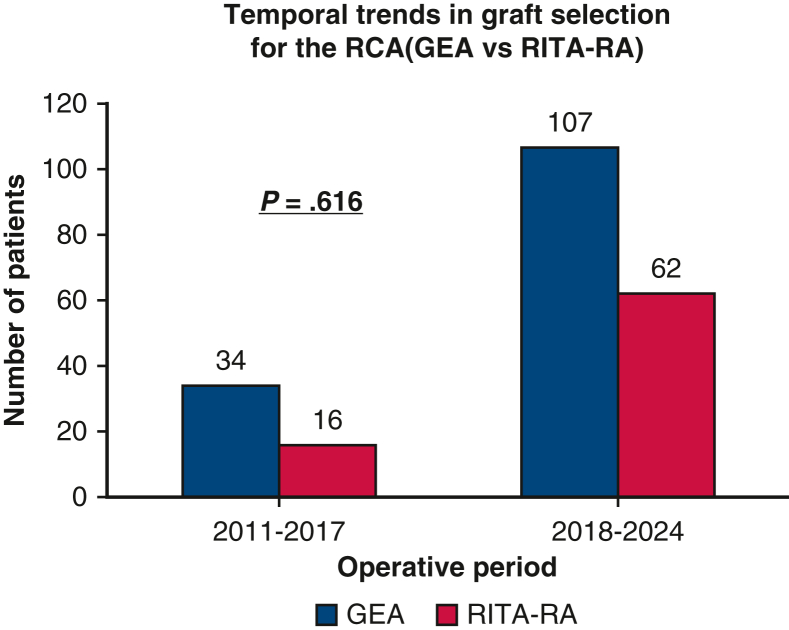
Temporal trends in graft selection for the right coronary artery (*RCA*) (gastroepiploic artery [*GEA*] vs right internal thoracic artery-radial artery [*RITA-RA*]). The number of patients in each period was 50 during 2011-2017 and 169 during 2018-2024, and no significant temporal bias was observed in graft selection for the RCA (*P* = .616).

Among 2527 patients who underwent isolated OPCAB at our center during the study period, 78 (53 [67.9%] men; mean age, 62.5 ± 8.7 years) and 141 (54 [38.3%] men; mean age, 64.1 ± 7.2 years) underwent RITA-RA and GEA grafting, respectively. The number of patients in each period was 50 during 2011-2017 and 169 during 2018-2024, and no significant temporal bias was observed in graft selection for the RCA (*P* = .616) (Figure 2).

After PSM, the RITA-RA and GEA grafting groups each comprised 65 patients (RITA-RA: 44 [67.7%] men, mean age 62.6 ± 9.4 years; GEA: 40 [61.5%] men, mean age 63.4 ± 6.7 years).

The preoperative patient characteristics are shown in Table 1. Before PSM, more patients in the RITA-RA group required preoperative IABP. Almost all baseline standardized mean differences were <0.15 after matching, with CKD slightly above, at 0.154.

**Table 1:** Preoperative patient characteristics before and after propensity score matching

Preoperative characteristics	Entire cohort			Matched cohort		
	RITA-RA (n = 78)	GEA (n = 141)	SMD	RITA-RA (n = 65)	GEA (n = 65)	SMD
Age (y)	62.5 ± 8.7	64.1 ± 7.2	.203	62.6 ± 9.4	63.4 ± 6.7	.092
Male sex	53 (67.9)	54 (38.3)	.131	44 (67.7)	40 (61.5)	.129
STS score	1.46 [7.55]	1.44 [14.83]	.217	1.46 [7.55]	1.21 [7.02]	.119
EuroSCORE Ⅱ	1.38 [11.05]	1.60 [19.33]	.308	1.45 [11.05]	1.30 [8.01]	.070
Comorbidity						
Hypertension	74 (94.9)	135 (95.7)	.041	63 (96.9)	62 (95.4)	.080
Diabetes mellitus	36 (46.2)	73 (51.8)	.113	32 (49.2)	31 (47.7)	.031
Dyslipidemia	72 (92.3)	137 (87.2)	.219	62 (95.4)	62 (95.4)	<.001
Chronic kidney disease∗	7 (9.0)	20 (14.2)	.163	8 (10.8)	5 (7.7)	.154
COPD	13 (16.7)	14 (9.9)	.199	11 (16.9)	11 (16.9)	<.001
PAD	9 (11.5)	17 (12.1)	.016	8 (12.3)	8 (12.3)	<.001
STEMI	14 (17.9)	16 (11.3)	.188	12 (18.5)	9 (13.8)	.126
Triple vessel disease	72 (92.3)	128 (90.8)	.055	60 (92.3)	61 (93.8)	.061
Preoperative PCI	3 (3.8)	11 (7.8)	.169	3 (4.6)	4 (6.2)	.068
Preoperative IABP	17 (21.8)	8 (5.7)	.482	6 (9.2)	6 (9.2)	<.001
Echocardiography						
Ejection fraction (%)	52.3 ± 15.1	52.5 ± 16.3	.022	52.4 ± 14.6	54.3 ± 14.3	.121
Urgency						
Elective	62 (79.5)	112 (79.4)	.001	54 (83.1)	56 (86.2)	.085
Urgent	16 (20.5)	29 (20.6)	.001	11 (16.9)	9 (13.8)	.085

Values are presented as mean ± SD, n (%), or median [interquartile range]. *RITA-RA*, Right internal thoracic and radial arteries; *GEA*, gastroepiploic artery; *SMD*, standardized mean difference; *STS*, Society of Thoracic Surgeons; *EuroSCORE*, European System for Cardiac Operative Risk Evaluation; *COPD*, chronic obstructive pulmonary disease; *PAD*, peripheral arterial disease; *STEMI*, ST-segment elevation myocardial infarction; *PCI*, percutaneous coronary intervention; *IABP*, intra-aortic balloon pump.

∗ Creatinine ≥1.5 mg/dL.

Table 2 presents the intraoperative data for both groups. After matching, the median number of graft conduits was significantly lower in the GEA group than in the RITA-RA group (median, 3 [range, 3-4] vs 3 [range, 2-4], respectively; *P* = .004). The total arterial revascularization rates were similar between groups and exceeded 90% (60 [92.3%] vs 61 [93.8%]; *P* = 1.000). The use of bilateral internal thoracic artery (BITA) for the left coronary artery (LCA) territory was more common in the GEA group; however, the rate of multiple arterial grafts use for the LCA, including the combination of a single ITA and a RA, was similar between the groups. Nongraft-related factors, such as operation time, NAT rate, and left minithoracotomy, did not differ between the groups.

**Table 2:** Perioperative details

Perioperative details	Entire cohort			Matched cohort		
	RITA-RA (n = 78)	GEA (n = 141)	*P* value	RITA-RA (n = 65)	GEA (n = 65)	*P* value
Operating time (min)	281.5 ± 61.5	258.0 ± 53.9	.004	279.7 ± 60.6	264.5 ± 51.5	.145
No. of distal anastomoses	3.0 [3.0]	3.0 [5.0]	.721	3.0 [3.0]	3.0 [3.0]	.929
Endarterectomy	2 (2.6)	0	.126	2 (3.1)	0	.496
Complete revascularization	78 (100.0)	139 (98.6)	.539	65 (100.0)	64 (98.5)	1.000
Conversion to CPB	0	0	NA	0	0	NA
No-touch aorta	76 (97.4)	135 (95.7)	.715	64 (98.5)	60 (92.3)	.221
Left mini-thoracotomy	0	8 (5.7)	.053	0	1 (1.5)	1.000
Graft						
LITA	78 (100)	137 (97.2)	.300	65 (100)	63 (96.9)	.496
RITA	78 (100)	117 (83.0)	<.001	65 (100)	55 (84.6)	.001
Radial artery	78 (100)	6 (4.3)	<.001	65 (100)	3 (4.6)	<.001
Gastroepiploic artery	0	141 (100)	<.001	0	65 (100)	<.001
Saphenous vein graft	7 (9.0)	7 (5.0)	.551	5 (7.7)	4 (6.2)	1.000
Total arterial revascularization	71 (91.0)	134 (95.0)	.260	60 (92.3)	61 (93.8)	1.000
BITA for LCA territory	1 (1.3)	117 (83.0)	<.001	0	54 (83.1)	<.001
MAG for LCA territory	66 (84.6)	121 (85.8)	.843	54 (83.1)	56 (86.2)	.809
No. of graft conduits	3.0 [1.0]	3.0 [3.0]	<.001	3.0 [1.0]	3.0 [2.0]	.002

Values are presented as mean ± SD, n (%), or median [interquartile range]. *RITA*-*RA*, Right internal thoracic and radial arteries; *GEA*, gastroepiploic artery; *CPB*, cardiopulmonary bypass; *NA*, not applicable; *LITA*, left internal thoracic artery; *RITA*, right internal thoracic artery; *BITA*, bilateral internal thoracic artery; *LCA*, left coronary artery; *MAG*, multiple arterial grafts.

Postoperative short-term outcomes are presented in Table 3. In the matched cohort, the duration of intensive care unit and in-hospital stays, major complications, and 30-day mortality did not differ between the groups. However, the rate of early extubation was higher in the RITA-RA group than in the GEA group (96.9% vs 84.6%; *P* = .030).

**Table 3:** Postoperative short-term outcomes

Postoperative outcomes	Entire cohort			Matched cohort		
	RITA-RA (n = 78)	GEA (n = 141)	*P* value	RITA-RA (n = 65)	GEA (n = 65)	*P* value
ICU stay (d)	2.0 [10]	2.0 [6.0]	.216	2.0 [10.0]	1.0 [5.0]	.676
In-hospital stay (d)	5.0 [16.0]	5.0 [18.0]	.257	5.0 [16.0]	5.0 [18.0]	.987
Early extubation (≤24 h)	75 (96.2)	125 (88.7)	.078	63 (96.9)	55 (84.6)	.043
Perioperative transfusion	55 (70.5)	94 (66.7)	.650	47 (72.3)	45 (69.2)	.85
Drain contents (mL)	476.9 ± 166.0	457.7 ± 164.1	.412	477.7 ± 175.6	467.1 ± 149.1	.662
30-d mortality	0	1 (0.7)	1.000	0	0	NA
Early-term postoperative complications						
New stroke	1 (1.3)	0	.356	1 (1.5)	0	1.000
New dialysis	0	0	NA	0	0	1.000
New onset atrial fibrillation/flutter	17 (21.8)	27 (19.1)	.725	14 (21.5)	11 (16.9)	.677
Wound infection	1 (1.3)	2 (1.4)	1.000	1 (1.5)	0	1.000
Reoperation for bleeding	0	2 (1.4)	.539	0	2 (3.1)	.496
Components of MACCE						
Cardiac death	2 (2.6)	8 (5.7)	.501	2 (3.1)	0	.496
Heart failure requiring hospitalization	3 (3.8)	0	.044	3 (4.6)	0	.244
Graft failure requiring reintervention	2 (2.6)	0	.356	1 (1.5)	0	1.000
Cerebral stroke and bleeding	3 (3.8)	3 (2.1)	.669	3 (4.6)	1 (1.5)	.619

Values are presented as mean ± SD, n (%), or median [interquartile range]. *RITA*-*RA*, Right internal thoracic and radial arteries; *GEA*, gastroepiploic artery; *ICU*, intensive care unit; *NA*, not applicable; *MACCE*, major adverse cardiac or cerebrovascular events.

**Figure 3:**
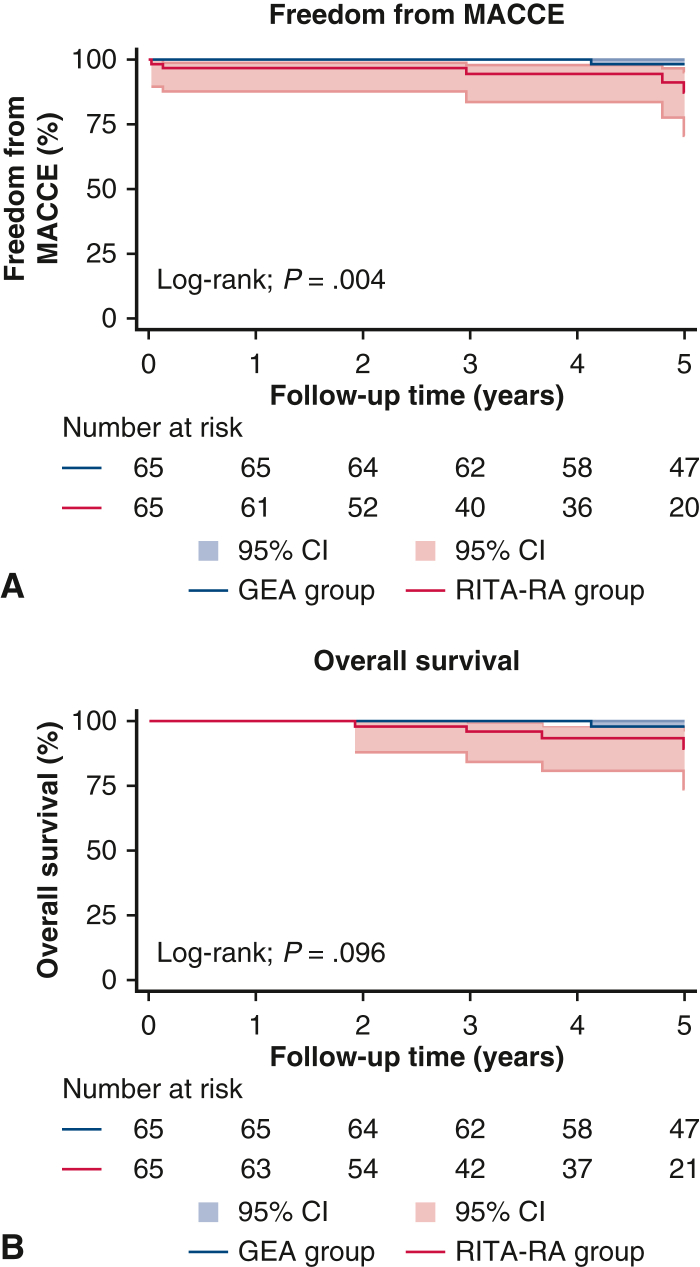
Kaplan-Meier analysis for freedom from major adverse cardiac or cerebrovascular event (*MACCE*) (A) and overall survival (B). Although the overall survival did not differ significantly between the 2 groups, the freedom from MACCE were significantly higher in the gastroepiploic artery (*GEA*) group. *RITA*-*RA*, Right internal thoracic artery-radial artery.

The long-term Kaplan-Meier curves of postoperative survival and freedom from MACCE are shown in Figure 3. The freedom from MACCE in the GEA group was better than that in the RITA-RA group (log-rank test *P* = .0041), particularly in heart failure requiring hospitalization (*P* = .040). However, the long-term overall survival did not differ significantly during a median follow-up of 5.0 years (*P* = .096). Among the components of MACCE, only heart failure requiring hospitalization occurred significantly more frequently in the RITA-RA group (log-rank test *P* = .041).

Table 4 shows the results of univariate and multivariate risk regression analyses to identify risk factors related to long-term MACCE and death. In univariate analysis, CKD, peripheral arterial disease (PAD), preoperative elective status, and postoperative new-onset atrial fibrillation/flutter were significant risk factors for both MACCE and death, whereas body mass index, preoperative IABP, and operation time were significant only for death. In multivariate regression analysis, a history of PAD (hazard ratio, 5.93; 95% CI, 2.11-16.7; *P* < .001) and preoperative IABP (hazard ratio, 3.85; 95% CI, 1.17-12.61; *P* = .026) remained independent predictors of long-term death. Regarding MACCE, only a history of PAD was a significant risk factor (hazard ratio, 3.68; 95% CI, 1.15-11.79; *P* = .028). In addition, the associations of arterial graft selection for the LCA—namely, BITA versus a single ITA and a RA—and the operating surgeon with both MACCE and overall death were assessed; however, neither was significantly associated with either outcome.

**Table 4:** Univariable and multivariable analyses for factors associated with long-term freedom from major adverse cardiac or cerebrovascular event (MACCE) and overall death

Variable	MACCE						Death					
	Univariable analysis			Multivariable analysis			Univariable analysis			Multivariable analysis		
	HR	95%CI	*P* value	HR	95%CI	*P* value	HR	95%CI	*P* value	HR	95%CI	*P* value
Preoperative factor												
Age	1.00	0.95-1.06	.981	–∗	–	–	1.05	1.00-1.10	.068	–	–	–
BMI	0.91	0.81-1.03	.139	–	–	–	.88	0.79-0.98	.025	0.88	0.77-1.00	.061
Male sex	0.47	0.19-1.18	.109	–	–	–	1.16	0.51-2.63	.727	–	–	–
Diabetes mellitus	2.21	0.84-5.85	.109	–	–	–	2.15	0.93-5.00	.074	–	–	–
Chronic kidney disease†	3.88	1.36-11.06	.011	2.15	0.67-6.90	.197	4.09	1.68-9.98	.0020	1.35	0.46-3.93	.578
COPD	1.26	0.37-4.32	.715	–	–	–	2.15	0.86-5.37	.103	–	–	–
PAD	6.13	2.33-16.14	<.001	3.68	1.15-11.9	.028	7.82	3.44-17.74	<.001	5.94	2.11-16.7	<.001
STEMI	–	–	–	–	–	–	0.19	0.025–1.43	.107	–	–	–
Triple vessel disease	0.77	0.18-3.344	.725	–	–	–	0.67	0.20-2.23	.510	–	–	–
Ejection fraction	0.18	0.0097-3.32	.249	–	–	–	0.12	0.0097-1.49	.100	–	–	–
Elective	0.31	0.12-0.77	.011	0.46	0.16-1.33	.150	0.24	0.11-0.52	<.001	0.60	0.22-1.66	.325
Preoperative IABP	2.39	0.78-7.33	.128	–	–	–	2.70	1.06-6.86	.037	3.85	1.17-12.61	.026
Intraoperative and postoperative factors												
Operation time	0.99	0.98-1.00	.193	–	–	–	0.99	0.98-0.99	.030	1.00	0.98-1.01	.437
Total arterial revascularization	1.45	0.19-10.99	.718	–	–	–	0.92	0.21-3.92	.905	–	–	–
New-onset atrial fibrillation/flutter	3.08	1.23-7.644	.016	1.49	0.50-4.46	.475	2.70	1.21-6.03	.015	0.77	0.26-2.27	.641
GEA graft	0.80	0.32-2.01	.641	0.82	0.16-4.34	.818	1.87	0.74-4.70	.184	0.989	0.21-4.64	.988
BITA for LCA territory	0.822	0.33-2.05	.675	0.88	0.21-3.64	.857	1.40	0.62-3.15	.417	1.38	0.43-4.45	.594
Single ITA and RA for LCA territory	0.68	0.22-2.06	.494	0.44	0.09-2.19	.317	0.21	0.05-0.89	.034	0.43	0.07-2.63	.367
Operating surgeon‡	0.89	0.31-2.51	.829	0.44	0.09-2.07	.297	8.23	1.11-61.1	.039	3.79	0.40-35.5	.243

*HR*, Hazard ratio; *BMI*, body mass index; *COPD*, chronic obstructive pulmonary disease; *PAD*, peripheral artery disease; *STEMI*, ST-segment elevation myocardial infarction; *IABP*, intra-aortic balloon pump; *GEA*, gastroepiploic artery; *BITA*, bilateral internal thoracic artery; *LCA*, left coronary artery; *ITA*, internal thoracic artery; *RA*, radial artery.

∗ Not included in the multivariable model; STEMI was not estimable due to no events.

† Creatinine ≥1.5 mg/dL.

‡ Dr Arayawudhikul versus others.

## Discussion

**Figure 4:**
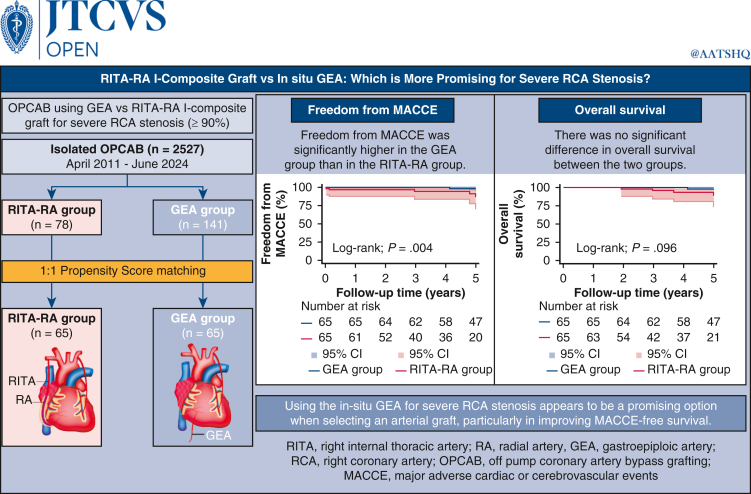
Graphical abstract. Each propensity score matched group comprised 65 patients. The freedom from major adverse cerebrovascular events (*MACCE*) in the gastroepiploic artery (*GEA*) group was better than that in the right internal thoracic artery-radial artery (*RITA-RA*) group.

The results of this study comparing surgical outcomes between RITA-RA I-composite and GEA grafts for RCA with >90% stenosis support the use of the GEA (Figure 4).

Satisfactory outcomes and usefulness of GEA for RCA have been widely reported. Nishida and colleagues^11^ reported excellent outcomes of CABG with only in situ bilateral ITA and GEA. Suzuki and colleagues^12^ also highlighted the superiority of skeletonized GEA grafting to the RCA over saphenous vein graft in left-sided bilateral ITA grafts. However, these outcomes require careful consideration of GEA use for the RCA. Yasuura and colleagues^13^ suggested a 0.5-mm larger GEA inner diameter than that of the RCA, and Nakajima and colleagues^14^ suggested using the GEA for proximal lesions to avoid low graft flow observed in distal lesions.

In this study, the minimum criteria for GEA use in the RCA were good GEA size based on intraoperative visual inspection and ≥90% RCA stenosis. However, further research on lesion location is needed. Moreover, when feasible, preoperative computed tomography scans may help improve medium-term results of in situ GEA grafts.^15^

Despite its advantages, using the GEA poses risks of subxiphoid hernias. Chan and colleagues^16^ identified subxiphoid hernias as a complication following median sternotomy, with long incisions such as those used when harvesting GEA grafts being a contributing factor. Although subxiphoid hernias is relatively rare, it is difficult to repair with recurrence rate. Although we have not encountered this complication, reports have prompted us to use the RITA-RA I-composite graft in certain cases. The results of the present study suggest that the using the GEA may be a promising option; however, subxiphoid hernias prevention measures, such as more robust suture closure, are required.

In this study, some of the outcomes of the I-composite graft comprising the RITA and RA were inferior to those of the GEA. Benedetto and colleagues^17^ observed graft kinking and stretching using the RITA as an in situ graft to the posterior descending artery, although RCA revascularization with the RITA was associated with superior late survival compared with the saphenous vein graft. To address the limited length of the RITA graft to the RCA, we have extended it using the RA, which is a useful conduit^18^, ^19^, ^20^; however, the outcomes have not been satisfactory. One possible reason is RA spasm. An in vitro study by Chardigny and colleagues^21^ found stronger vasoreactivity in the RA than in the GEA, which explains its higher propensity to spasm. Microscopically, the RA has a considerably thicker media containing a high density of smooth muscle cells compared with the ITA and GEA, making it more prone to spasm despite easier anastomosis.^22^ To address this, pharmacological agents, including phenoxybenzamine, an alpha-adrenoceptor antagonist, and nitroglycerin, can effectively prevent early-term RA spasms.^23^, ^24^, ^25^ Although late postoperative RA spasms are rare, calcium channel blockers like amlodipine and nifedipine can significantly reduce RA vasoconstriction induced by neurohormonal factors post-CABG.^26^ We routinely administer calcium channel blockers after surgery; however, in some cases, we refrain from administering them if the patient's blood pressure is low. Incorporating these treatments to the greatest extent possible may improve long-term patency.

Another potential issue is the length of the I-composite graft. A single RITA graft may be too short for distal RCA grafting, which could cause graft kinking and stretching. However, extending the RA can also lead to kinks or twists. Wise and colleagues^27^ suggested that grafts to the posterior branches of the right or circumflex coronary arteries are particularly prone to twisting and kinking due to their considerable length. Additionally, longer grafts should be avoided for other reasons. Chowdhury and colleagues^28^ reported that most RA conduits exhibited preexisting intimal hyperplasia, particularly in the distal portion, especially in elderly men and individuals with diabetes, who smoked, had hypertension, and PAD. Therefore, careful consideration of RA graft length is critical.

The rate of BITA use for the LCA territory, which was reported to influence long-term outcomes positively in some previous reports,^29^^,^^30^ was significantly higher in the GEA group. However, the rate of MAG, including the combination of a single ITA and a RA, was similar between the groups. The addition of either RITA or RA to a single ITA has been reported to improve long-term outcomes.^31^^,^^32^ In this study, the association between arterial graft selection for the LCA and long-term outcomes was also assessed, but no significant relationship with MACCE or overall survival was found. Therefore, the observed difference in MACCE between the groups is unlikely to be attributable to the difference in BITA use.

For NAT, we used the RITA-RA I-composite graft; however, the in situ RITA may not be the best inflow source for the RA. Jung and colleagues^33^ reported superior early and late patency rates of CABG using RA-to-aorta anastomosis compared with left ITA-RA composite grafts. Conversely, some studies recommend the in situ ITA as an inflow source for the RA. For instance, a series of seven case studies by Gatti and colleagues^34^ demonstrated favorable outcomes for the RITA-RA I-composite graft. This technique avoids aortic clamping and proximal aortic anastomoses, enables BITA use, prevents the RITA from crossing anteriorly to the heart, and reduces the risk of air embolism into the coronary tree. The only drawbacks were the need for good matching of size, thickness, and vessel wall quality between the proximal RITA and the RA graft and ensuring sufficient RA length to reach its coronary target without tension. Additionally, nitric oxide released from the endothelial cells in the ITA may act on the RA intimal layer within the I-composite graft, helping to prevent RA graft spasm.^35^^,^^36^ However, a lack of consensus warrants further research on this unique composite graft.

### Study Limitations

The study limitations include the retrospective, nonrandomized single-center design and the small number of patients. A major limitation of this study is the lack of graft patency data because we did not perform routine postoperative CT or coronary angiography. Because graft patency is a key determinant of long-term outcomes in patients undergoing CABG, the absence of these data limits our ability to directly assess the durability and efficacy of the grafts. This limitation may influence the interpretation of our findings, particularly regarding the long-term survival benefits associated with different graft choices.

## Conclusions

In OPCAB, the GEA appears to be a promising option for RCA with severe stenosis, particularly in improving MACCE-free survival. However, because graft patency data were not available in this study, further research is required to validate these findings and determine the optimal arterial graft for the RCA.

### Webcast

You can watch a Webcast of this AATS meeting presentation by going to: Xxx.

## Conflict of Interest Statement

The authors reported no conflicts of interest.

The *Journal* policy requires editors and reviewers to disclose conflicts of interest and to decline handling or reviewing manuscripts for which they may have a conflict of interest. The editors and reviewers of this article have no conflicts of interest.

## References

[1] Rocha R.V., Tam D.Y., Karkhanis R. (2018). Multiple arterial grafting is associated with better outcomes for coronary artery bypass grafting patients. Circulation.

[2] Rocha R.V., Tam D.Y., Karkhanis R. (2020). Long-term outcomes associated with total arterial revascularization vs non-total arterial revascularization. JAMA Cardiol.

[3] Tatoulis J., Wynne R., Skillington P.D., Buxton B.F. (2016). Total arterial revascularization: a superior strategy for diabetic patients who require coronary surgery. Ann Thorac Surg.

[4] Tatoulis J., Buxton B.F., Fuller J.A. (2011). The right internal thoracic artery: the forgotten conduit—5,766 patients and 991 angiograms. Ann Thorac Surg.

[5] Spadaccio C., Fremes S.E., Gaudino M.F.L. (2019). Right internal thoracic or radial artery as the second arterial conduit for coronary artery bypass surgery. Curr Opin Cardiol.

[6] Emmert M.Y., Seifert B., Wilhelm M., Grünenfelder J., Falk V., Salzberg S.P. (2011). Aortic no-touch technique makes the difference in off-pump coronary artery bypass grafting. J Thorac Cardiovasc Surg.

[7] Lev-Ran O., Braunstein R., Sharony R. (2005). No-touch aorta off-pump coronary surgery: the effect on stroke. J Thorac Cardiovasc Surg.

[8] Gülcan O., Türköz R., Demirturk O.S., Oguzkurt L., Türköz A. (2008). Extending the boundaries of no-touch aorta technique usage for coronary artery bypass grafting in patients with diseased ascending aorta. J Cardiovasc Surg (Torino).

[9] Ushioda R., Hirofuji A., Yoongtong D. (2024). Assessing the benefits of anaortic off-pump coronary artery bypass grafting. Front Cardiovasc Med.

[10] Kanda Y. (2013). Investigation of the freely available easy-to-use software “EZR” for medical statistics. Bone Marrow Transplant.

[11] Nishida H., Tomizawa Y., Endo M., Koyanagi H., Kasanuki H. (2001). Coronary artery bypass with only in situ bilateral internal thoracic arteries and right gastroepiploic artery. Circulation.

[12] Suzuki T., Asai T., Matsubayashi K. (2011). In off-pump surgery, skeletonized gastroepiploic artery is superior to saphenous vein in patients with bilateral internal thoracic arterial grafts. Ann Thorac Surg.

[13] Yasuura K., Takagi Y., Ohara Y., Takami Y., Matsuura A., Okamoto H. (2000). Theoretical analysis of right gastroepiploic artery grafting to right coronary artery. Ann Thorac Surg.

[14] Nakajima H., Takazawa A., Yoshitake A. (2019). Current mechanisms of low graft flow and conduit choice for the right coronary artery based on the severity of native coronary stenosis and myocardial flow demand. Gen Thorac Cardiovasc Surg.

[15] Yokoyama K., Yoshizaki T., Nagaoka E., Tasaki D., Arai H. (2023). Preoperative computed tomography of the right gastroepiploic artery for coronary artery bypass grafting. Circ J.

[16] Chan J., O’Hanlon J., McKenna J., Oo S. (2021). Subxiphoid incisional hernias post median sternotomy: a literature review. J Card Surg.

[17] Benedetto U., Caputo M., Gaudino M., Mariscalco G., Bryan A., Angelini G.D. (2017). Is the right internal thoracic artery superior to saphenous vein for grafting the right coronary artery? A propensity score–based analysis. J Thorac Cardiovasc Surg.

[18] Gaudino M., Benedetto U., Fremes S. (2018). Radial-artery or saphenous-vein grafts in coronary-artery bypass surgery. N Engl J Med.

[19] Cao C., Manganas C., Horton M. (2013). Angiographic outcomes of radial artery versus saphenous vein in coronary artery bypass graft surgery: a meta-analysis of randomized controlled trials. J Thorac Cardiovasc Surg.

[20] Tranbaugh R.F., Dimitrova K.R., Friedmann P. (2012). Coronary artery bypass grafting using the radial artery: clinical outcomes, patency, and need for reintervention. Circulation.

[21] Chardigny C., Jebara V.A., Acar C. (1993). Vasoreactivity of the radial artery. Comparison with the internal mammary and gastroepiploic arteries with implications for coronary artery surgery. Circulation.

[22] van Son J.A., Smedts F., Vincent J.G., van Lier H.J., Kubat K. (1990). Comparative anatomic studies of various arterial conduits for myocardial revascularization. J Thorac Cardiovasc Surg.

[23] Harrison W.E., Mellor A.J., Clark J., Singer D.R. (2001). Vasodilator pre-treatment of human radial arteries; comparison of effects of phenoxybenzamine vs papaverine on norepinephrine-induced contraction in vitro. Eur Heart J.

[24] Taggart D.P., Dipp M., Mussa S., Nye P.C. (2000). Phenoxybenzamine prevents spasm in radial artery conduits for coronary artery bypass grafting. J Thorac Cardiovasc Surg.

[25] Shapira O.M., Alkon J.D., Macron D.S. (2000). Nitroglycerin is preferable to diltiazem for prevention of coronary bypass conduit spasm. Ann Thorac Surg.

[26] Bond B.R., Zellner J.L., Dorman B.H. (2000). Differential effects of calcium channel antagonists in the amelioration of radial artery vasospasm. Ann Thorac Surg.

[27] Wise E.S., Cheung-Flynn J., Brophy C.M. (2016). Standard surgical skin markers should be avoided for intraoperative vein graft marking during cardiac and peripheral bypass operations. Front Surg.

[28] Chowdhury U.K., Airan B., Mishra P.K. (2004). Histopathology and morphometry of radial artery conduits: basic study and clinical application. Ann Thorac Surg.

[29] Bakaeen F.G., Ravichandren K., Blackstone E.H. (2020). Coronary artery target selection and survival after bilateral internal thoracic artery grafting. J Am Coll Cardiol.

[30] Schwann T.A., Hashim S.W., Badour S. (1997). Improved survival with multiple left-sided bilateral internal thoracic artery grafts. Ann Thorac Surg.

[31] Schwann T.A., Hashim S.W., Badour S. (2016). Equipoise between radial artery and right internal thoracic artery as the second arterial conduit in left internal thoracic artery-based coronary artery bypass graft surgery: a multi-institutional study. Eur J Cardiothorac Surg.

[32] Tranbaugh R.F., Dimitrova K.R., Lucido D.J. (2014). The second-best arterial graft: a propensity analysis of the radial artery versus the free right internal thoracic artery to bypass the circumflex coronary artery. J Thorac Cardiovasc Surg.

[33] Jung S.H., Song H., Choo S.J. (2009). Comparison of radial artery patency according to proximal anastomosis site: direct aorta to radial artery anastomosis is superior to radial artery composite grafting. J Thorac Cardiovasc Surg.

[34] Gatti G., Taffarello P., De Groodt J., Benussi B. (2020). A non-conventional proximal inflow for the radial artery coronary graft. Interact Cardiovasc Thorac Surg.

[35] Tanyeli O., Duman I., Dereli Y., Gormus N., Toy H., Sahin A.S. (2019). Relaxation matters: comparison of in-vitro vasodilatory role of botulinum toxin-A and papaverine in human radial artery grafts. J Cardiothorac Surg.

[36] Ueda T., Taniguchi S., Kawata T., Mizuguchi K., Nakajima M., Yoshioka A. (2003). Does skeletonization compromise the integrity of internal thoracic artery grafts?. Ann Thorac Surg.

